# Supplementary figures and images for: Duplication, Divergence and Cardiac Expression of Tropoelastin in Jawed Fishes, Including Tetraploid Rainbow Trout (Oncorhynchus mykiss)
Source: Genes (Basel). 2025 Dec 13;16(12):1492. doi: 10.3390/genes16121492 (PMC12733013; doi:10.3390/genes16121492)

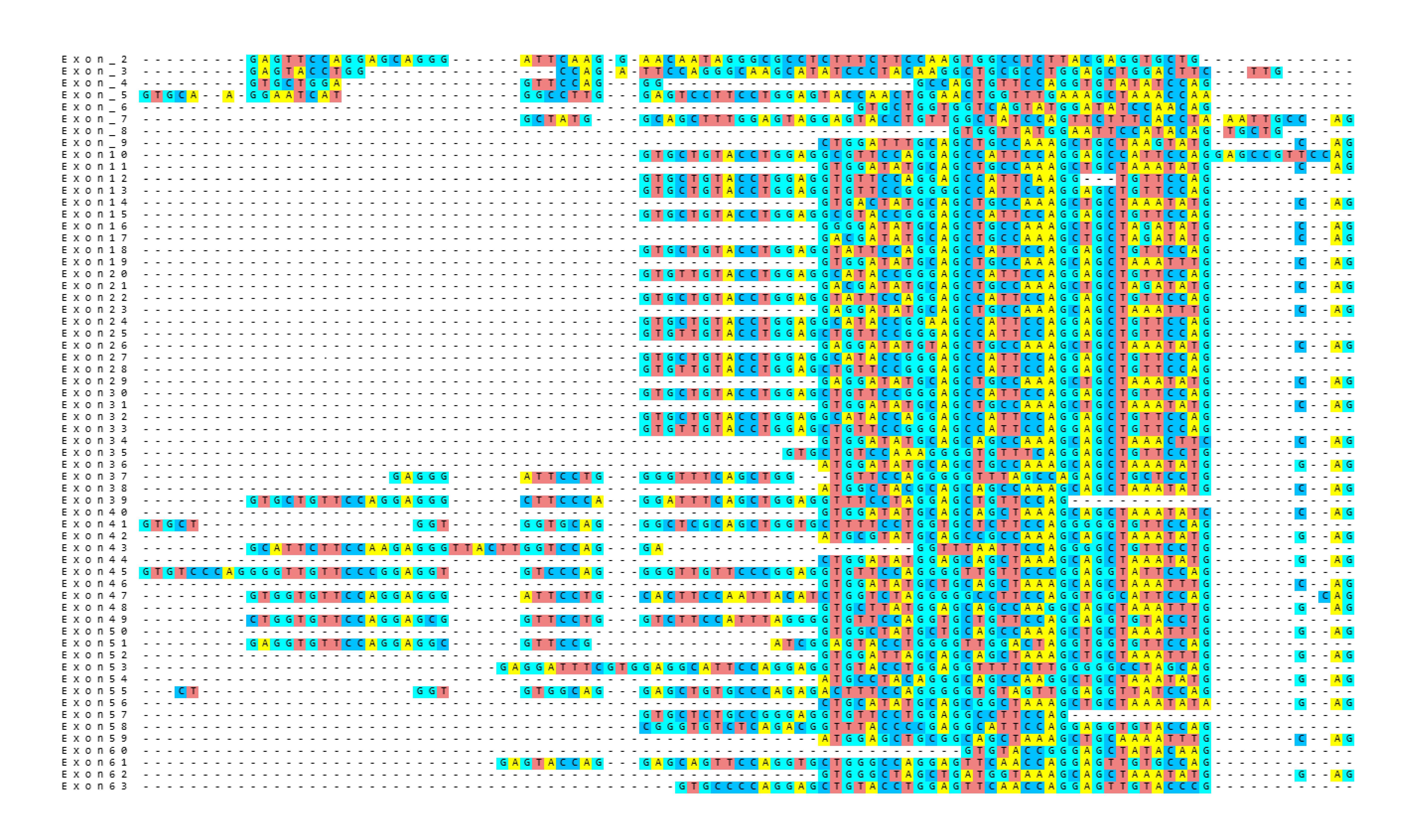

Supplement: Supplementary file 1 [file genes-16-01492-s001.zip › Figure S1 - Final.tif]

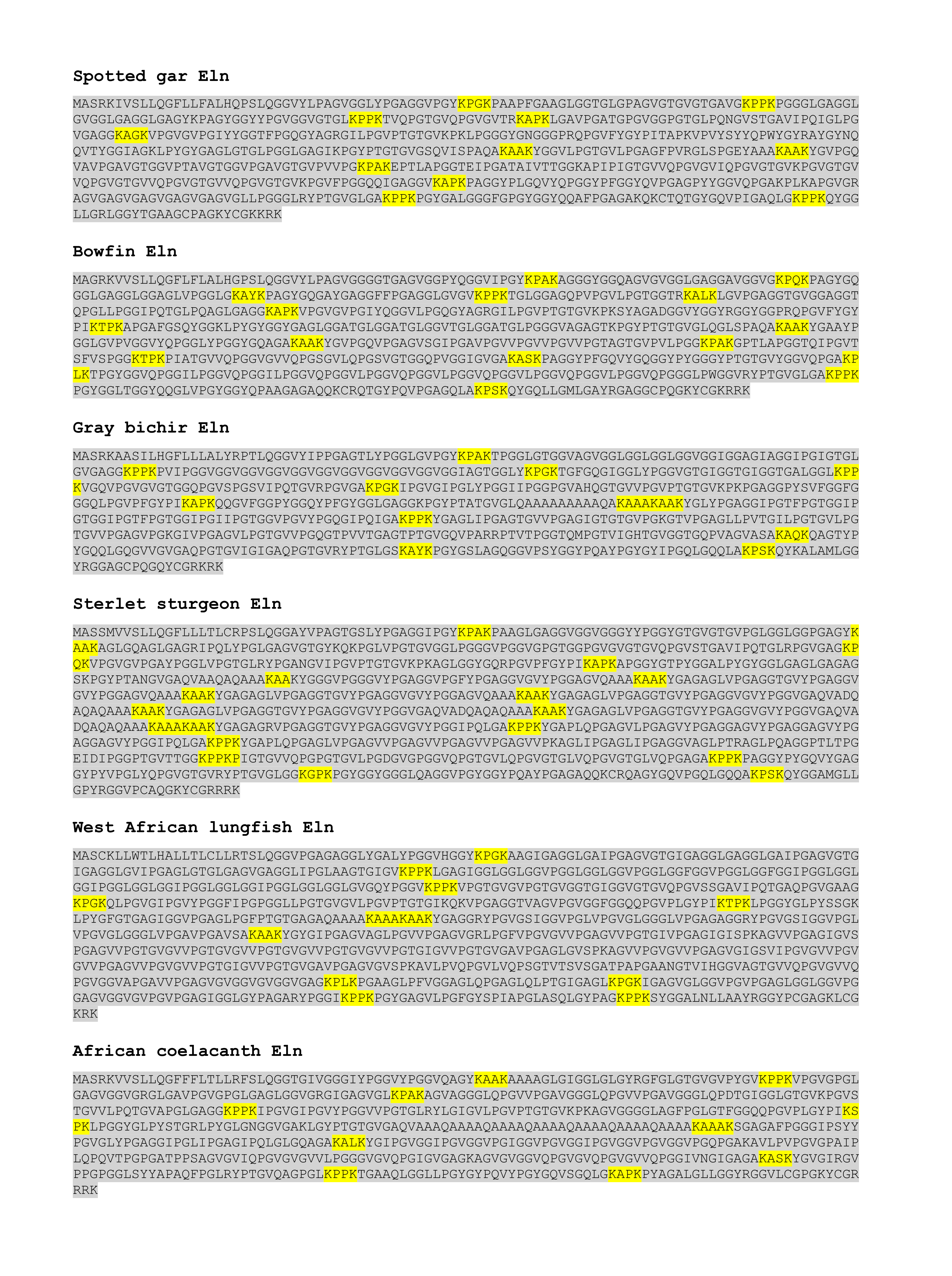

Supplement: Supplementary file 1 [file genes-16-01492-s001.zip › Figure S2 - Final.tif]

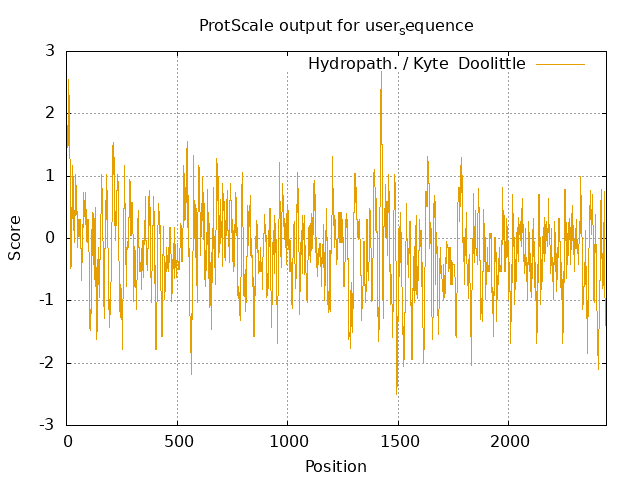

Supplement: Supplementary file 1 [file genes-16-01492-s001.zip › Figure S3_revised.gif]
